# Matlock House Hydropathic Establishment

**Published:** 1902-01-11

**Authors:** 


### MATLOCK HOUSE HYDROPATHIC ESTABLISHMENT.

THREE hours and a half after leaving St. Pancras the traveller may arrive at Matlock Bridge, and find himself amid the beautiful scenery of Derbyshire. Hardly anywhere in England do the hills lay themselves out in more graceful outline, and somewhere in this neighbourhood the tired or sick man may readily venture to rest until health and strength come to him again. Crossing the bridge just outside the little station, he is soon at the foot of a steep hill up which a road or street runs in an almost straight line, and over which a cable-tram will pull him up if the hill frighten him.

On a crest of this hill, known as Matlock Bank, and commanding extensive views in almost every direction, stands the Matlock House Hydropathic Establishment—a building in the Italian Palazzo style of architecture. It is large enough to contain nearly 100 patients and visitors. The site is about 700 feet above the sea-level: the building faces the south-west, and it is considerably sheltered from the north-east winds, a matter of some importance at this altitude. The grounds belonging to the institution are not very extensive, but they are tastefully laid out in shrubberies, lawns and tennis grounds. At the present time alterations are in progress to make the surroundings more attractive; but as the patient has the whole of the scenery of Matlock, of this "gem of the Peak," within easy reach, the immediate neighbourhood is, perhaps, not of very great moment. So much for the outside of the institution. Inside we find a large hall or dining-room capable of seating 80 or 100 people. A fixed stage is placed at one end for theatrical performances and concerts. There are many private sitting rooms and a good drawing-room and lounge.

Dr. Moxon is physician to the establishment, and it is his aim to give it a more distinctly medical element than is common in such institutions. To promote this he has recently fitted up a complete installation of the Dowsing electric plant for the radiant heat and electric light treatment of disease. There are three methods of application in use here. One is a bedstead with asbestos coverings and containing 10 Dowsing lamps. In this the whole body, except the head, can be subjected to radiant heat and electric light, and a temperature of 300° is easily maintained. If ever required it could be considerably increased; and one of the most striking and valuable features of this form of Turkish bath is the extreme ease with which the temperature can be increased, lowered, or maintained. Here, too, the heat is unmistakably and directly radiant, and it was long since discovered that this radiant heat was greatly to be preferred to heated air conducted in pipes to the hot-room of an ordinary Turkish bath. There are arrangements by which two Dowsing lamps can be used locally for any limb or part of a limb, and another in which the radiant heat and light of a powerful lamp can be concentrated on any part of the head or neck.

The treatment seems very beneficial in some forms of rheumatoid arthritis, chronic rheumatism and chorea, peripheral neuritis, spinal diseases, etc. Dr. Sibley, of the North-West London Hospital, says that many more diseases than might at first sight be imagined are benefited by radiant heat, and he adds that his "rule at the hospital for some years has been, when a patient suffering from any complaint does not improve after a course of routine drug-treatment, to order radiant heat, and in the majority of cases improvement very quickly shows itself, often to a marked degree." This may savour rather of optimism; but that benefit often follows is undoubted.

At Matlock House the usual baths found in all hydropathic establishments are in use, and it is worth noting that the bath-rooms are of modern construction, and that those for ladies are entirely separated from those for men. Brine baths can be had, and massage and the Nauheim treatment for heart disease carried out. The terms are from £2 2s. to £4 4s. a week. Medical attendance and some of the baths are extras. The hours are—breakfast, 8.30; luncheon, 1; and dinner, 7. Judging from the menus submitted to us we should describe the diet as both liberal and varied.

To those unacquainted with Derbyshire, it may be mentioned that in the neighbourhood of Matlock Bridge are Haddon Hall, Baslow, Chatsworth, Buxton, Willasley Castle, Circle of Arbor Low, Taddington, the highest village in England, Ruins of Wingfield Manor, etc.

